# Case report: CMV retinitis following local and systemic immunosuppression

**DOI:** 10.3389/fopht.2024.1354104

**Published:** 2024-03-06

**Authors:** Bryant A. Menke, William Wagner, Helen Song, William Thomas, Natalia E. Castillo Almeida, Christopher D. Conrady, Steven Yeh

**Affiliations:** ^1^ Department of Ophthalmology, Truhlsen Eye Institute, University of Nebraska Medical Center, Omaha, NE, United States; ^2^ Cole Eye Institute, Cleveland Clinic, Cleveland, Ohio, OH, United States; ^3^ Omaha Eye and Laser Institute, Omaha, NE, United States; ^4^ Division of Infectious Disease, University of Nebraska Medical Center, Omaha, NE, United States; ^5^ National Strategic Research Institute, University of Nebraska Medical Center, Omaha, NE, United States; ^6^ Global Center for Health Security, University of Nebraska Medical Center, Omaha, NE, United States

**Keywords:** cytomegalovirus, retinitis, intravitreal dexamethasone, immunosuppression, case report

## Abstract

We report the case of a 71-year-old man who presented 2 years following renal transplantation with diffuse, unilateral cytomegalovirus retinitis five weeks after receiving an intravitreal dexamethasone implant device for the management of central retinal vein occlusion. Examination of the left eye showed diffuse retinal hemorrhages, attenuated and tortuous retinal vessels, and superior retinal whitening. The patient was successfully treated with serial intravitreal foscarnet injections and oral valganciclovir with disease regression observed by 12 weeks after presentation. The patient’s visual acuity and examination remained stable at 9-months follow-up.

## 1 Introduction

Cytomegalovirus (CMV) retinitis is a vision-threatening infection most commonly seen in immunocompromised patients, but also rarely seen in immunocompetent patients, particularly if exposed to local corticosteroid therapy (1). Three patterns of CMV retinitis have been previously characterized: fulminant/hemorrhagic, granular, and frosted branch angiitis (2). The fulminant pattern of CMV retinitis presents as an extensive area of hemorrhagic necrosis admixed with retinal edema and white/yellow cloudy retinal lesions (3). The granular pattern presents as peripheral retinal white/cloudy lesions with minimal necrosis or hemorrhage (3, 4). The frosted branch angiitis pattern classically shows white lesions surrounding retinal vessels (4). The diagnosis of CMV retinitis may be made clinically. However, polymerase chain reaction (PCR) of aqueous or vitreous fluid may be utilized to confirm the diagnosis, follow treatment response, and detect the presence or absence of antiviral resistance mutations (5, 6).

CMV retinitis is one of the most common ocular opportunistic infections seen in transplant patients who are chronically immunocompromised. Local corticosteroid administration in the setting of systemic immunosuppression may further increase a patient’s risk of developing CMV retinitis. Intravitreal dexamethasone is clinically indicated for patients with non-infectious posterior uveitis, retinal vein occlusion, and diabetic retinopathy complicated by macular edema (7). Cases of CMV retinitis following implantation have been reported previously but are infrequent (8–12). Here, we present the case of a renal transplant patient on systemic immunosuppression who developed CMV retinitis five weeks after implantation of an intravitreal dexamethasone implant for a longstanding central retinal vein occlusion. Risk factors for CMV retinitis included the patient’s history of CMV viremia, long-term immunosuppression following renal transplantation, and local corticosteroid exposure.

## 2 Case description

A 71-year-old male was referred to our clinic for anterior chamber inflammation in the left eye of one-week duration that had not responded to prednisolone 1% drops four times per day, and new retinal whitening following intravitreal dexamethasone 0.7 mg (Ozurdex®, AbbVie, North Chicago, IL) performed for a central retinal vein occlusion five weeks prior to referral. Of note, the patient had received multiple anti-VEGF injections in the past, but due to persistent macular edema, intravitreal dexamethasone implant was previously used. The patient had received multiple intravitreal dexamethasone implants for 5 years prior to this episode.

His current medications included mycophenolate 1440 mg daily, belatacept infusions monthly, and oral prednisone 5 mg daily.

Ophthalmic history was notable for central retinal vein occlusion (CRVO) diagnosed in the left eye four years prior and CRVO of the right eye two years prior. The patient underwent a kidney transplant two years prior to presentation. The donor was CMV-positive and the patient was CMV-negative. Nine months following transplantation, he developed CMV viremia with a quantitative viral level of 264,000 IU/mL (CMV Log10 of 5.42). He was treated with oral valganciclovir 900 mg twice daily for two months, at which point he was titrated up to 900 mg every day for another month. He then developed drug-induced neutropenia from valganciclovir and was monitored for serum CMV DNA on a weekly basis.

**Figure 1:**
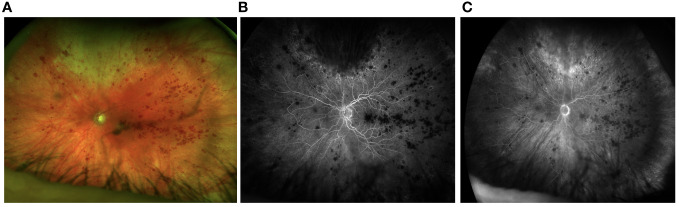
Fundus photos from initial presentation shows active retinitis superiorly **(A)**. Fluorescein angiography shows delayed perfusion and hypoperfusion of temporal retina **(B)** with mild hyperfluorescence of the disc in the late phases of the angiogram. The perfusion delay persists **(C)**.

The patient reported reduced vision in the left eye. His visual acuity was measured at 20/200 OS and 20/30 OD. Examination revealed 1+ cell in the anterior chamber, and an intravitreal dexamethasone implant cast within the vitreous cavity. Fundus examination was significant for subfoveal hemorrhage and diffuse intraretinal hemorrhage. The retinal vessels were attenuated and tortuous. Retinal whitening was observed superiorly with extension to the equator and retinitis was also observed inferotemporally. Fluorescein angiography showed leakage near foci of retinitis superiorly, hyperfluorescence of the disc, and diffuse peripheral retinal ischemia (**Figure 1**).

## 3 Diagnostic evaluation and management

An anterior chamber (AC) tap was performed, and the aqueous sample was sent for PCR for Varicella Zoster Virus (VZV), Herpes Simplex Virus (HSV), CMV, and Toxoplasmosis. Empiric treatment of 900 mg valganciclovir twice per day and intravitreal foscarnet 2.4 mg/0.1 cc were administered for suspected viral retinitis.

PCR testing was positive for CMV and quantitative serum level of CMV was 593 IU/mL. The dosing of valganciclovir was co-managed with the nephrology and transplant infectious diseases team. A second dose of intravitreal foscarnet was administered three days after the presentation. Eight days following the initial presentation, a third intravitreal foscarnet injection was performed, and an aqueous sample was obtained and sent for antiviral drug resistance mutation testing. The testing, specifically assessing for UL54 CMV DNA polymerase and UL97 protein kinase mutations, was negative for resistance genes. Intravitreal foscarnet injections were subsequently continued weekly for four weeks, and prednisolone drops were tapered to once daily.

**Figure 2:**
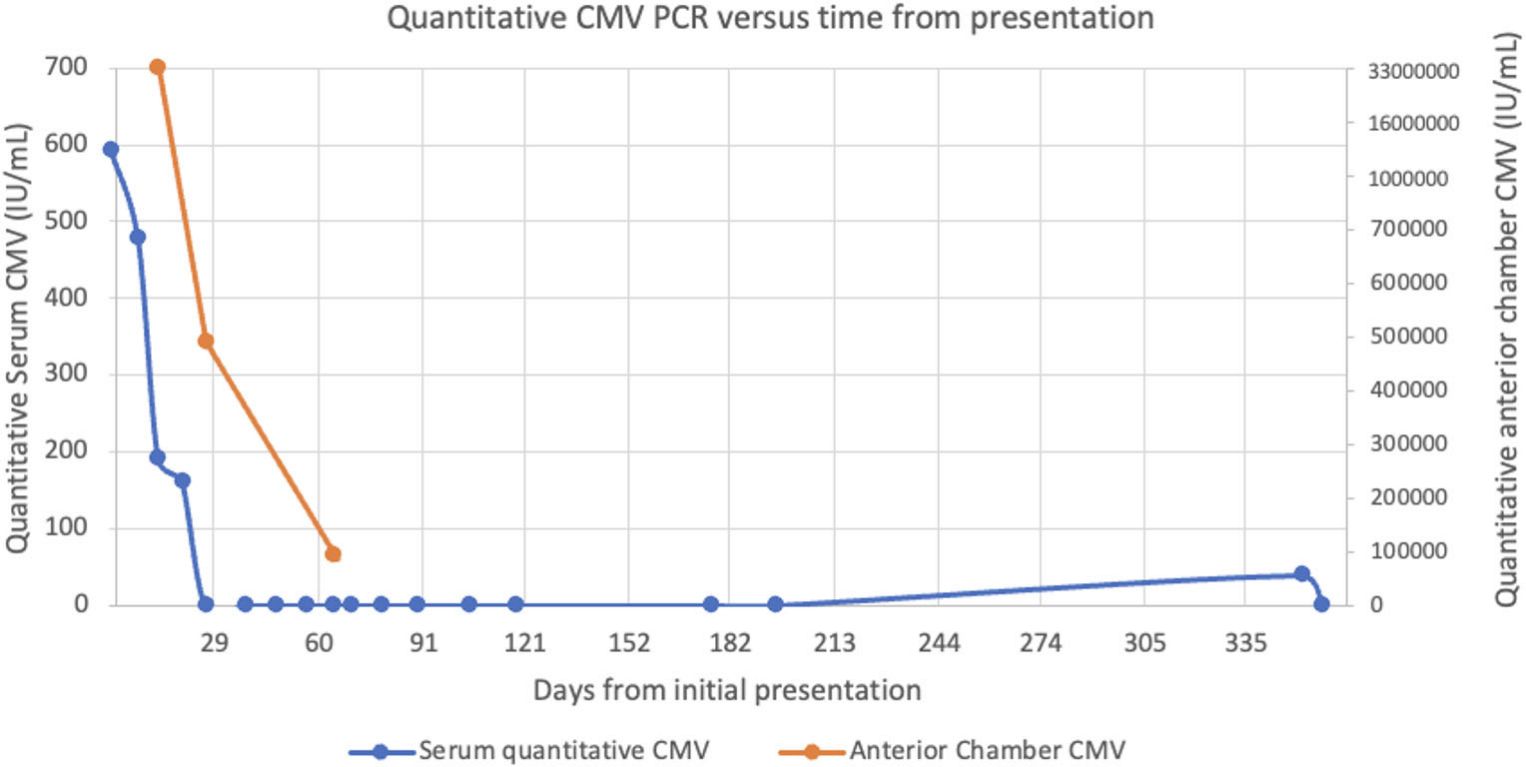
Plot showing the relationship between quantitative serum and anterior chamber CMV PCR values relative to the time from initial presentation.

**Figure 3:**
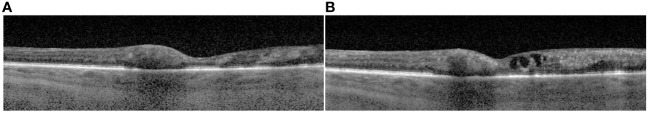
Macular optical coherence tomography (mOCT) images from the day of initial presentation **(A)** and 7 weeks following initial presentation prior to aflibercept injection where there is slight thickening of the macula due to intraretinal fluid **(B)**.

Our patient was noted to have poor optic nerve perfusion and serial AC paracenteses were performed to reduce intraocular pressure and avoid an ischemic event. These samples were sent to quantitate the intraocular viral load. The quantitative AC CMV PCR declined from 33 million IU/mL after the first injection to 490 thousand IU/mL after the fifth injection and 92 thousand IU/mL after the seventh injection, respectively. Paired serum CMV DNA was notably undetectable during this time. A chart showing serum and AC quantitative CMV PCR is shown in **Figure 2**. An aflibercept injection 2 mg/0.05 (Regeneron, Tarrytown, NY, USA) was administered seven weeks after initial presentation due to slightly increased retinal thickening on macular optical coherence tomography (mOCT) in **Figure 3**.

**Figure 4:**
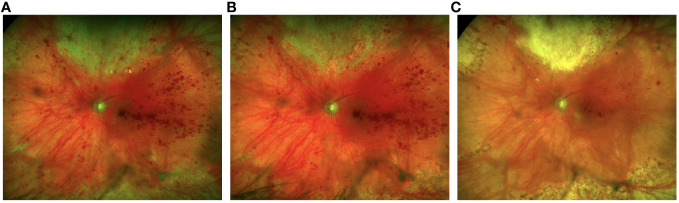
Fundus photos at 2 weeks following 3 intravitreal foscarnet injections shows improvement of the retinitis superiorly **(A)** with continued resolution at 1 month following a total of 5 intravitreal foscarnet injections **(B)**. There is complete resolution of the retinitis with fibrosis superiorly and reduced retinal heme at 1-year follow-up **(C)**.

By 12 weeks post-presentation, no active retinitis was present. At this time, the patient’s best-corrected visual acuity was 20/150 in the left eye. After nine months, valganciclovir was discontinued given his clinically stable retinal disease. **Figure 4** shows fundus photos from initial presentation to the most recent exam, two years following initial presentation.

## 4 Discussion

CMV retinitis is a vision-threatening condition that typically occurs due to the reactivation of CMV, most often in an immunocompromised host. CMV retinitis can present with acute onset floaters, blurred vision, and/or new-onset blind spots in the vision. It can become vision-threatening through direct damage to the retina and optic nerve or due to a retinal detachment, which may occur in as many as 50% of patients (13, 14). A funduscopic exam is critical for diagnosing suspected patients and demonstrates dense retinal whitening and retinitis with irregular borders that typically follows the vasculature (15). Scattered satellite lesions are also a classic feature of the disease (16). The diagnosis does not require microbiological confirmation of aqueous or vitreous fluid if the clinical picture is characteristic (3).

CMV retinitis typically occurs in immunocompromised patients including patients with HIV, but the prevalence has reduced with the widespread use of anti-retroviral therapy. Immunocompromised patients who may remain at risk for CMV retinitis include post-transplant patients, patients with systemic autoimmune disease on immunomodulatory therapy, and has also been described in patients following local corticosteroid injections (1, 5, 15). In renal transplant patients, optimizing the level of immunosuppression to prevent opportunistic infections is critical (16). Renal transplant patients considered high risk are started on prophylactic or preemptive antiviral therapy (17). In patients who are negative for CMV serologies and receive a CMV-positive allograft, as in our patient, 56% develop CMV disease without such preventative treatment (18). In these patients, administering prophylactic valganciclovir, intravenous ganciclovir, or valacyclovir is recommended (19). However, antiviral drug resistance mutations may develop in patients treated with long-term prophylactic valganciclovir and may be detected through aqueous humor samples (20).

Up to 12% of patients with recipient-negative, donor-positive kidney transplants have ganciclovir-resistant CMV (21). The most common mutations are of the UL97 gene, which codes for a kinase that phosphorylates ganciclovir, or the UL54 gene, which codes for viral DNA polymerase. Screening for CMV resistance should be considered in select populations, especially those on chronic valganciclovir therapy or refractory CMV retinitis cases (20).

Our patient was clinically stable on his immunomodulatory therapy until five weeks after receiving an intravitreal dexamethasone implant device. As he only manifested ocular symptoms, we suspect the intraocular steroid treatment combined with his systemic immunomodulating therapy may have increased the risk for CMV reactivation. Given serum CMV DNA was <600 IU/mL and soon became undetectable, serial aqueous humor CMV testing and ophthalmic exam guided the management as aqueous humor CMV DNA levels are related to CMV retinitis disease activity (22).

A review of the literature revealed five reported cases of CMV retinitis following the implantation of intravitreal dexamethasone devices. Three of these cases occurred in immunocompetent patients – one occurred in an older man postulated to be at risk for reactivation due to immunosenescence (8–10). One of the reported cases was a patient who received systemic immunomodulatory therapy for the management of CRVO due to retinal vasculitis and subsequently developed retinal detachment and light perception vision in the affected eye, underscoring the vision-threatening nature of this infectious retinitis (11).

One other case reported of CMV retinitis following an intravitreal dexamethasone implant in a patient on immunomodulatory therapy for a kidney transplant has been reported (12). This occurred two years post kidney transplantation and developed symptoms of retinitis eight weeks following device implantation. This patient was managed with intravitreal and systemic ganciclovir with improved VA and retinitis nine months after treatment initiation.

As novel immunomodulatory therapies for autoimmune disease and immunosuppression for solid organ and hematologic transplant recipients continue to evolve, opportunistic infections for patients at risk of CMV require ongoing surveillance. Our patient’s prior history of CMV viremia, combined with the need for systemic immunosuppression and local corticosteroid for a retinal vein occlusion, demonstrated the multifactorial risk that may have contributed to the development of CMV retinitis. Given the differences in the clinical phenotype of non-HIV-related immunosuppression from HIV patients, further studies in CMV retinitis in this patient population are needed.

## Data availability statement

The original contributions presented in the study are included in the article/supplementary material. Further inquiries can be directed to the corresponding author.

## Ethics statement

Ethical approval was not required for the study involving humans in accordance with the local legislation and institutional requirements. Written informed consent to participate in this study was not required from the participants or the participants’ legal guardians/next of kin in accordance with the national legislation and the institutional requirements. Written informed consent was obtained from the individual(s) for the publication of any potentially identifiable images or data included in this article.

## Author contributions

BM: Investigation, Writing – original draft, Writing – review & editing. WW: Writing – review & editing. HS: Writing – review & editing. WT: Writing – review & editing. NA: Writing – review & editing. CC: Writing – review & editing. SY: Investigation, Supervision, Writing – review & editing.
